# Ductus Arteriosus in Fetal and Perinatal Life

**DOI:** 10.3390/jcdd11040113

**Published:** 2024-04-01

**Authors:** Flaminia Pugnaloni, Daniela Doni, Mariella Lucente, Stefano Fiocchi, Irma Capolupo

**Affiliations:** 1 Neonatal Intensive Care Unit, Fetal Neonatal and Cardiological Science Research Area, “Bambino Gesù” Children’s Hospital, IRCCS, 00165 Rome, Italy; flaminia.pugnaloni@opbg.net; 2 Neonatal Intensive Care Unit, Fondazione IRCCS San Gerardo Dei Tintori, 20900 Monza, Italy; danieladoni9@gmail.com; 3 Neonatal Intensive Care Unit, Azienda Ospedaliera di Cosenza, 87100 Cosenza, Italy; lucentem7@gmail.com; 4 Pediatric Department, G. Fornaroli Hospital, 20013 Magenta, Italy; stefanofiocchi66@gmail.com

**Keywords:** patent ductus arteriosus, fetus, newborn, premature babies

## Abstract

The ductus arteriosus represents an essential vascular structure connecting the pulmonary artery and the aorta. Over the past decades, there has been substantial advancement in our understanding of both the fundamental and clinical aspects of the ductus arteriosus. In particular, the clarification of the regulatory mechanisms governing ductal patency in critical stages such as the fetal and the perinatal period has enabled optimal management of both physiological and pathological conditions in which the ductus arteriosus plays a crucial role. Furthermore, a more in-depth understanding of the regulatory mechanisms controlling this fundamental structure has facilitated the development of advanced therapeutic strategies and personalized interventions. In the present review, we provide a comprehensive overview of the ductus arteriosus during fetal and perinatal life, encompassing its physiological functions, pathological conditions, and clinical implications. Through this examination, we aim to contribute to a broader understanding of the ductus arteriosus’ role in these critical developmental stages and its significance in clinical practice.

## 1. Introduction

The ductus arteriosus (DA) is an important fetal structure connecting the pulmonary and systemic circulations, allowing for fetal blood to bypass the developing lungs. Despite centuries of study, the DA’s complexity still represents a great challenge for both clinicians and scientists.

Early anatomical descriptions date back to the second century AD, with Sir William Harvey recognizing its importance in fetal circulation in the 1600s [1]. Seminal studies in the following centuries detailed the DA’s histological structure [2,3] while others provided mechanistic insights into the involvement (or role) of prostaglandin and oxygen in regulating the patency and closure of the DA [4,5]. Recent advances in molecular biology and diagnostics have enhanced our understanding, but several issues persist, in particular regarding the genetic predisposition to persistent DA, the timing of spontaneous closure, and optimal pharmacologic strategies [6].

This review explores several factors influencing DA patency during fetal and perinatal life.

## 2. Ductus Arteriosus in Fetal Life

### 2.1. Embryology, Morphology, and Histology

In humans, the ductus arteriosus is a crucial vascular structure fully formed between the sixth and eighth weeks of gestation [7].

In fetuses with normal cardiac anatomy, the DA connects the main pulmonary artery to the descending aorta, usually distal to the origin of the left subclavian artery.

Embryologically, the DA arises from the distal portion of the left sixth pharyngeal arch approximately at day 29 of fetal life and creates stable communication between the dorsal aorta and the pulmonary trunk subsequent to the emergence of the right and left pulmonary arteries from the proximal end of the sixth branchial arch. Rarely, a right DA may arise due to a regression failure of the right sixth arch [8]. An important study has taken into account the spatial relationship between the fetal DA and the aorta because the location of DA insertion defines the inferior margin of the aortic isthmus [7].

The ductus arteriosus plays a pivotal role in fetal circulation by establishing a connection between the main arteries, and the dimensions and configuration of the ductus arteriosus significantly influence the resistance to blood flow and the extent of shunting [9].

Moreover, the magnitude and direction of the flow across the ductus arteriosus (DA) determine that the upstream angle between the DA and the aorta is typically <90°, while the downstream angle between the DA and the descending aorta is usually ≥80° [10]. This configuration is referred to as the “F type” or “fetal type” in the classification proposed by Krichenko and colleagues [11]. The term “fetal-type” DA therefore describes a distinctive elongated ductus with angles resembling a hockey stick at its insertion point into the pulmonary artery [12].

During gestation, the DA becomes progressively longer, wider, and more curved, and by the end of the third trimester, it will exhibit a pronouncedly curved configuration [13].

From the histological point of view, there are significant differences between the great arteries (i.e., the main pulmonary artery and descending aorta) and DA. The (tunica) media of the DA is indeed characterized by smooth muscle fibers with a typical longitudinal and spiral arrangement, whereas the media of the great arteries is mainly composed of circumferentially arranged layers of elastic fibers. The contraction of the smooth muscle fibers of the media is responsible for the progressive narrowing of the DA, starting at the pulmonary end of this structure and leading to the functional closure within the first days of life in term newborns [9,13]. Moreover, the DA intima is thick and irregular, and during the third trimester, it is characterized by the presence of intraluminal intimal cushions [14]. This distinctive structure of the ductus arteriosus lays the foundation for its constriction following birth.

The patency of the DA is controlled by opposing mechanisms that induce vasodilation or vasoconstriction. During fetal life, it is crucial for the DA to remain open as a result of a predominance of vasodilatory factors over vasoconstrictive ones. Prostaglandins, in particular, play a pivotal role in preventing preterm vasoconstriction. Histological changes during ductal maturation result in increased responsiveness to oxygen tension and prostaglandins [15].

Circulating prostaglandins, which originate in the placenta, usually undergo metabolism in the lungs. Because fetal pulmonary blood flow is low, the clearance of prostaglandins is diminished, resulting in an elevated concentration of these factors [16].

Fetal DA patency is predominantly maintained through prostaglandin E2 (PGE2) activation, facilitated by the EP4 receptor, inducing DA dilation via the activation of the cAMP/PKA signaling pathway [17].

Simultaneously, endothelial nitric oxide synthase produces nitric oxide (NO) in the luminal endothelium and vasa vasorum endothelium. The activation of nitric oxide (NO), which stimulates the cGMP/PKG signaling pathway, is fundamental to maintaining fetal ductal patency [6].

While PGE2 and NO are commonly recognized as the principal mediators of DA dilation, it has been recently demonstrated that other factors also play a significant role. Adenosine and atrial natriuretic peptide have been documented to dilate the DA by upregulating cAMP and cGMP signaling, respectively [18]. Furthermore, several potassium channels expressed in smooth muscle cells (SMCs) function as vasodilators along with carbon monoxide (CO), which serves as a cGMP-mediated vasodilator [19].

All these molecular mechanisms are required to maintain DA patency in the fetal life [20,21].

Human genetic studies have unveiled several genetic factors linked to both syndromic and nonsyndromic cases of the PDA. Currently, over 100 disorders encompass the PDA within their phenotypic spectrum, supporting the idea that several genes can contribute to syndromic PDA. Notable syndromic examples encompass Down syndrome, Marfan syndrome, and Noonan syndrome [22]. Conversely, nonsyndromic cases occur in isolation and exhibit a higher prevalence compared to syndromic associations [23,24]. Additionally, research utilizing mouse knockout models has provided valuable insights into the molecular pathways involved in PDA development and the genetic background [25].

### 2.2. Ductus Arteriosus Physiology in Fetal Life

Before birth, fetal hemodynamics are characterized by low blood flow in pulmonary circulation (functional absence of the lungs) and a dependence on low-resistance placental circulation for metabolic functioning as well as gas exchange.

The majority of our current knowledge of the physiology of fetal circulation is based on findings from animal models [10,25]. Fetal blood distribution deeply differs from neonatal circulation because the blood flow of the intrauterine circulation system is directed to maximize oxygen delivery to the brain and heart. In the fetus, blood is oxygenated in the placenta and returns to the body through the umbilical vein. About 50% of oxygenated blood flow, coming from the placenta, reaches the inferior vena cava via the ductus venosus and about 50% is distributed to the hepatic circulation system. Umbilical venous blood has an oxygen saturation of 70% to 80%, which is the highest oxygen saturation in fetal circulation [10,26].

Due to the anatomical position of the inferior vena cava and its connection with the atria, the majority of the oxygen-rich blood volume exhibits a preferential flow pattern into the left atrium via the patent foramen ovale (PFO). The maintenance of FO patency is due to a pressure gradient between the two atria, which allows for the unidirectional flow of blood into the left atrium and subsequently the left ventricle. A significant portion of this oxygen-enriched blood, characterized by an oxygen saturation of 65%, is directed from the ascending aorta toward the upper regions of the fetal body, with a smaller fraction directed to the descending aorta [26].

The remaining blood within the right atrium mixes with blood from the superior vena cava prior to entering the right ventricle. Subsequently, it is expelled into the pulmonary trunk, with an average oxygen saturation level of 50%. Because a notable feature in fetal circulation is the elevated pulmonary vascular resistance (PVR), only a fraction of about 20% of the right ventricular output traverses the pulmonary circulation [26].

The majority of the right ventricular output is directed through the ductus arteriosus and reaches the limited blood supply originating from the ascending aorta. Consequently, the final oxygen saturation level within the blood in the descending aorta is approximately 55%.

#### Inizio Modulo

Animal studies and Doppler studies in human fetuses have demonstrated that almost 80–90% of the blood volume that is pumped into the pulmonary artery by the right fetal ventricle preferentially joins the systemic flow via the ductus arteriosus (DA) and, as a result, only a percentage of 10–20% of the right cardiac output enters the lungs during this period [6].

DA patency is therefore essential to ensure fetal development.

### 2.3. Echographic Imaging

In a typical fetal ultrasound, an axial three-vessel trachea view reveals the pulmonary artery connecting with the ductal arch, positioned on the same side as the aorta in a V-shaped alignment relative to the trachea. This view also includes a visible superior vena cava. When observing the ductal and aortic arch views in a sagittal orientation, it becomes evident that the aortic arch has a sharper bend and originates more posteriorly from the left ventricle than the ductal arch, which has its origin in the right ventricle. The three-vessel and trachea view is becoming a routine assessment in fetal scans [9].

Fetal ductal flow is entirely right to left during the cardiac cycle and it is typically biphasic, characterized by a peak systolic velocity up to 140 cm/s and diastolic velocities ranging from 6 to 30 cm/s [27].

### 2.4. Fetal Ductus Arteriosus Pathology

Understanding the physiological effect of the DA during fetal life is essential in comprehending the normal development of the cardiovascular system and the potential anomalies that can arise from a defective DA.

An intrauterine constrictive DA is a rare pathological entity mostly occurring during the third trimester of gestation. The risk of ductal constriction shows, in fact, a significant increase with advancing gestational age, especially from 32 weeks of gestation [28].

Ductal constriction is often associated with maternal intake of prostaglandin synthase inhibitors (non-steroidal anti-inflammatory drugs or corticosteroids) or the consumption of foods rich in polyphenols (green tea, dark chocolate, and grape juice) [29].

A prenatal constrictive DA determines significant hemodynamic alteration mostly driven by higher pulmonary vascular resistance, leading to early fetal right ventricular (RV) failure, increased pulmonary blood flow, and consequent microstructural changes in the pulmonary vascular bed and pulmonary arteries dilation [30].

Fetal echocardiographic studies revealed a small diameter of the DA, high systolic and diastolic Doppler velocities, and a decreased pulsatility index. Moreover, additional findings may include RV hypertrophy with/without dysfunction, tricuspid and pulmonary regurgitation, right atrium dilation, and cardiomegaly.

Recent studies confirmed that fetal hearts with DA constriction show significant ventricular remodeling and biventricular dysfunction through conventional ultrasound parameters [31,32].

Most severe cases of DA constriction may result in hydrops fetalis and fetal demise [28,33].

The prompt discontinuation of causative agent exposure can result in the normalization of the ductal flow in the majority of cases, whereas the detection of complete prenatal ductal closure with or without hydrops currently represents an indication for urgent delivery in order to avoid prenatal death and further damage to the fetal pulmonary bed [28].

Postnatal persistent pulmonary hypertension of the newborn (PPHN) may occur in almost 25% of patients with in utero DA constriction and requires global management of pulmonary vascular resistance (nitric oxide, supplementary oxygen, and mechanical ventilation) and pharmacological management of cardiac dysfunction.

## 3. Ductus Arteriosus in the Transitional Period

The transition from intrauterine to extrauterine life represents a critical phase with rapidly evolving circulatory changes, which may have downstream end-organ consequences.

Animal models have been used to assess the physiological processes typical of the transition phase with particular effort in characterizing the hemodynamic changes during this period [34].

The complex transition of fetal circulation to postnatal hemodynamic status is a result of a multilevel interplay among biochemical, mechanical, and hormonal factors that are still not fully elucidated and, thus, are currently under investigation [16,17,35,36,37].

The main trigger for these complex changes after birth is aeration of the lungs secondary to the onset of spontaneous breathing. The negative pressure created by the first breaths is the cause of the displacement of lung fluid from the alveoli to the interstitial space and establishment of an air–liquid interface with the subsequent aeration of the lungs, increasing the pO_2_ and causing a significant drop in PVR [34].

Over the first 48–72 h of life, a progressive fall in PVR secondary to progressive lung recruitment and increased alveolar oxygen content has been demonstrated [34].

Concurrently, the removal of placental circulation through cord clamping determines a rise in SVR and a rapid shift of the PVR:SVR ratio with preferential shunting from systemic-to-pulmonary circulation (i.e., left-to-right shunting) across the DA and FO. The DA left-to-right shunting triggers a significant rise in the blood volume into the pulmonary vascular bed with the following exposure of the pulmonary endothelium to increased shearing forces.

Simultaneously, increasing oxygen tension across the DA leads to several biochemical changes. In particular, there is significant production of vasodilatory mediators (i.e., nitric oxide and prostacyclin) and reduced production of vasoconstrictor mediators (i.e., endothelin, leukotrienes, and thromboxane) [38,39].

Therefore, the shunt across the DA during the transition plays a critical role in driving hemodynamic changes during this vulnerable period.

Closure of the DA occurs spontaneously after birth in most infants, and it transforms into the “ligamentum arteriosum”, a fibrous band of tissue that is a remnant of the DA. However, the mechanism regulating DA closure is complex and multifactorial. Postnatal closure of the DA can be categorized into two phases: an early functional closure followed by an anatomical closure.

The DA’s functional closure is mostly driven by smooth muscle cell (SMC) constriction secondary to increased arterial oxygen content, a prostaglandin concentration fall, and decreased blood pressure (due to the drop in PVR). Functional closure of the lumen of the DA occurs within the first 18–24 h after birth in full-term healthy neonates, whereas the anatomical closure takes days or weeks to be completed.

The anatomical closure depends on several molecules that have been recently reviewed in a study by Hsu et al. [37]. In particular, several remodeling mechanisms, including SMC migration/proliferation, extracellular matrix (ECM) production, endothelial cell (EC) proliferation, and internal elastic laminae (IEL) disruption, significantly contribute to the anatomical closure of the DA [37].

**Figure 1:**
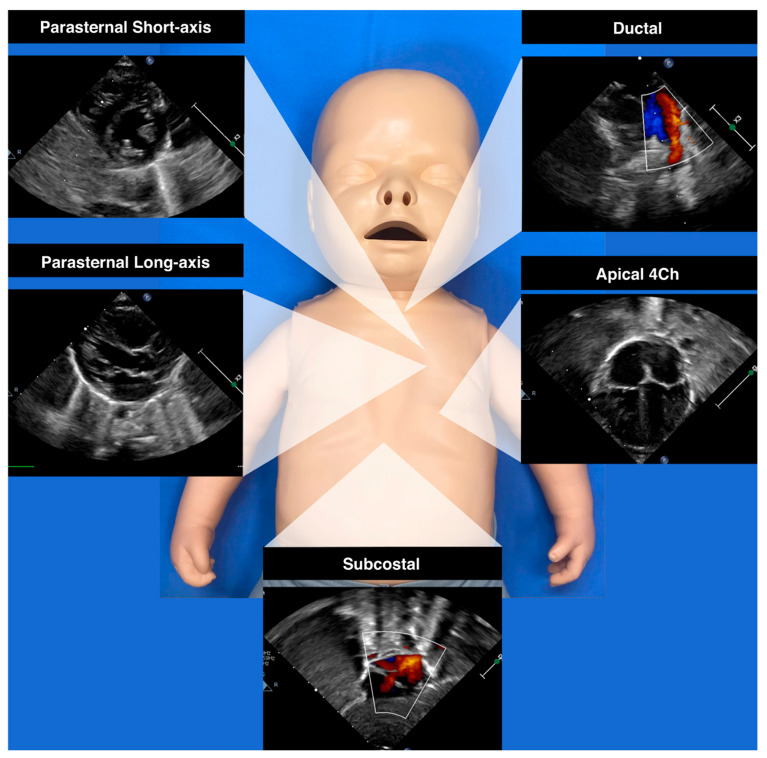
Standard echocardiographic views used in NPE.

The growing application of neonatologist-performed echocardiography (NPE) and functional echocardiographic techniques allowed for the early detection of postnatal DA changes in neonates [40,41]. Figure 1 provides a concise visual representation of the key imaging perspectives employed in the evaluation of ductal shunts and cardiac chambers. Patterns of ductal shunting in a normal trajectory have been described in a prospective study by Jain et al. and it has been demonstrated that right-to-left DA flow beyond 8–12 h of life is uncommon for full-term healthy neonates [42].

Nevertheless, how the ductal shunt may be affected by several factors that potentially interfere with a normal transition warrants further evaluation.

### Ductus Arteriosus during Transition in Premature Preterm Neonates

The DA in preterm neonates is less likely to close during the transitional period and, indeed, the incidence of patent ductus arteriosus (PDA) is strongly related to lower gestational ages. PDA in preterm infants, moreover, is strongly associated with several complications, such as chronic lung disease (CLD) and other adverse outcomes [43,44].

The DA in preterm infants shows some histological differences because it is more thin-walled and less muscular. Moreover, the premature ductus’ SMCs appear to be more sensitive to circulating PGE2 and nitric oxide, whose levels are higher when preterm due to systemic inflammatory mediators (Tumor necrosis factor alpha, TNFα) [45].

Preterm babies also show a variable degree of adrenal insufficiency with altered cortisol secretion and a subsequent higher risk of prolonged DA patency [46]. Additional factors that may be significantly associated with premature DA patency are thrombocytopenia [47] and altered platelet function [48].

Impaired O_2_ responsiveness in the DA promotes persistent patency, and the oxygen-sensing mechanism has been the object of several studies to date [20,49,50]. The recent application of multiomic approaches to biological processes has gained a new insight into our understanding of the DA oxygen response.

Transcriptomic studies performed in animal models and human samples identified several genes that are differentially expressed in the DA and are important for DA function and the response to O_2_ (i.e., TFAP2B and prostaglandin E2 receptor PTGER4) [51]. Transcriptomics also revealed significantly different SMC expression profiles in cases of a normal DA, a closing pattern DA, and PDA. In patients with a closing DA, there was a significant enrichment of the gene ontology pathways to the oxygen response. Omic strategies (transcriptomics and proteomics) share the potentiality to enhance our understanding of the complexity of gene and protein expression implicated in the mechanism of closure of the DA [52].

NPE studies have also taken into consideration the potential role of resuscitation techniques including positive pressure ventilation and oxygen and surfactant administration on the patency of the DA.

Surfactant replacement therapy can promote a major circulatory shift, favoring pulmonary circulation (at the expense of systemic circulation) through the PDA. Using serial echocardiography, Sehgal et al. demonstrated changes in the transductal diameter, flow direction, and shunt magnitude after surfactant replacement therapy in newly born preterm infants [53].

Early exposure to high-flow systemic-to-pulmonary shunting across the DA during the transitional period in preterm neonates may have an etiological role for peculiar complications in this population.

Relevant complications occurring in the immediate transitional period are mostly represented by intraventricular hemorrhage (IVH) and pulmonary hemorrhage (PH).

The premature myocardium is characterized by a lower number of contractile units and an immature sarcoplasmic reticulum [54]. Clinically, this translates to a slower adaptation to preload and afterload changes typical of the transitional period. In particular, a sustained systemic-to-pulmonary shunt across a premature DA determines a sudden increase in the pulmonary blood flow, pulmonary venous return, and left heart preload. However, the premature left ventricle may not tolerate these changes and compensatory mechanisms may include increased left ventricular relaxation and filling, increased left atrial pressure, and postcapillary pulmonary hypertension, thus increasing the preterm’s susceptibility to complications, such as pulmonary hemorrhage [53].

**Figure 2:**
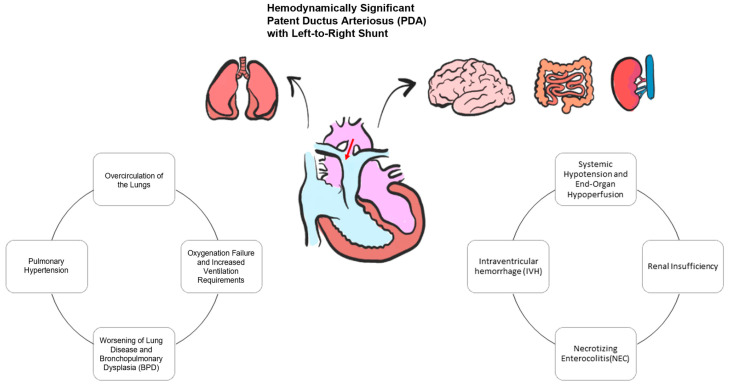
Hemodynamically significant patent ductus arteriosus pathophysiology.

Additionally, sustained prolonged unrestrictive left-to-right shunting across the DA can lead to a shunting of blood away from vital postductal organs, resulting in compromised perfusion to these critical tissues and a steal phenomenon downstream, including cerebral and systemic circulation. In some cases, the extent of the DA shunt may supersede the infant’s compensatory mechanisms of increasing left ventricular output (cerebral autoregulation capacity), thus resulting in a variable degree of cerebral hypoperfusion, which may predispose one to IVH [55] (Figure 2).

Several studies have tried to analyze the association between preterm PDA and short-term and long-term outcomes [56].

However, a recent multicenter trial has concluded that expectant management for PDA in extremely premature infants was noninferior to early ibuprofen treatment concerning outcomes such as necrotizing enterocolitis, bronchopulmonary dysplasia, or mortality at 36 weeks postmenstrual age. This pivotal study paves the way for a potential shift in the approach to PDA in preterm neonates [57].

In a population-based cohort of extremely preterm infants, the practice of early echocardiographic evaluation within the first 3 days of life and DA treatment was significantly associated with both lower mortality and a lower incidence of PH [58].

To date, however, the ability to detect ductal physiological changes during transition has been hindered by the absence of equipment capable of continuously measuring hemodynamic changes. NPE allows for the periodic evaluation of the presence, shunt direction, and shunt volume of the DA in preterm infants, but there is still the need for the validation of the association between these echocardiographic parameters and the outcome.

The patency of the DA may also be a therapeutic option in specific pathological entities.

In particular, maintaining DA patency by prostaglandin infusion is the therapeutic cornerstone in the case of duct-dependent congenital heart defects (Critical CHD) until a surgical or interventional repair can be provided. These conditions include both duct-dependent CHD affecting the systemic circulation (hypoplastic left heart syndrome, hypertrophic cardiomyopathy with critical left ventricular output impairment, critical aortic stenosis, and coarctation of the aorta or interrupted aortic arch) and duct-dependent CHD affecting the pulmonary circulation (tricuspid valve atresia, severe Ebstein’s anomaly, pulmonary atresia, critical pulmonary artery stenosis, and severe tetralogy of Fallot).

Moreover, an extracardiac malformation may benefit from ductal patency as this important vascular structure may preserve ventricular function by “offloading” pulmonary circulation (i.e., Vein of Galen malformation and congenital diaphragmatic hernia-related pulmonary hypertension).

In all these conditions, the therapeutic effect of DA patency is largely based on maintaining the balance between systemic blood flow (Qs) and pulmonary blood flow (Qp). With a normal hemodynamic status, without intracardiac or extracardiac shunts, an equal volume of blood flows through the pulmonary and systemic vascular beds, resulting in a Qp:Qs ratio of 1. In several conditions where there is the need for ductal patency, the main aim is to keep the Qp:Qs ratio within a “safe range” through routine surveillance of the patient’s symptoms indicative of low or excessive blood flow.

NPE can be used to estimate the left and right ventricular outputs and extrapolate the Qp:Qs ratio; despite being readily available for bedside monitoring, a reference method is currently lacking, and it lacks reliability in the case of outflow tract obstruction and in the case of PDA bidirectional shunting.

## 4. Conclusions

The ductus arteriosus plays a key role both during fetal life and in the transitional period.

Proper knowledge of the status of the DA shunt in fetuses, preterm, and at-term babies is important for a full evaluation of the hemodynamic changes over time.

After birth, a DA shunt may contribute to the pathophysiology of hemodynamic complications and, on the other hand, in pathological conditions, ductal patency could be necessary to ensure adequate pulmonary and/or systemic blood flow.

Close monitoring through bedside and non-invasive tools such as NPE is fundamental for an early assessment of the quantitative and qualitative changes in the DA shunt and to identify newborns at higher risk for several pathological conditions.

Despite the greater availability of advanced molecular biology techniques and substantial progress in diagnostics and patient global care, the patent ductus arteriosus (PDA) continues to raise important issues regarding the genetic and epigenetic factors contributing to PDA, the optimal timing for spontaneous closure, and the pharmacological approaches for managing a persistently patent DA.

## 5. Future Directions

Advancements in the comprehension of regulatory mechanisms and the physiopathology of the fetal and perinatal ductus arteriosus represent a crucial step in the field of neonatal research. Elucidating the intricate determinants governing ductus arteriosus patency during these critical developmental stages holds significant implications for both basic science and clinical care. Future directions in this field necessitate a comprehensive integration of high-throughput omics technologies, such as genomics, transcriptomics, and proteomics, to elucidate the complex interplay of molecular factors. Collaborative efforts of molecular biologists, bioinformaticians, and clinicians are essential for the synthesis of a comprehensive understanding. This approach will allow for a deeper understanding of the regulatory networks, providing a foundation for targeted therapeutic interventions and innovative strategies to address the multiple issues related to DA function in the fetal and perinatal periods.

## Data Availability

Dataset available on request from the authors.
